# How to mitigate the risks of deployment of artificial intelligence in medicine?

**DOI:** 10.55730/1300-0144.5814

**Published:** 2024-05-20

**Authors:** Sevil UYGUN İLİKHAN, Mahmut ÖZER, Hande TANBERKAN, Veysel BOZKURT

**Affiliations:** 1 Department of Internal Medicine Sciences, Gülhane Faculty of Medicine, University of Health Sciences, Ankara, Turkiye; 2 Commission of National Education, Culture, Youth and Sports of the Parliament, Ankara, Turkiye; 3 Presidency of Strategy and Budget, Ankara, Turkiye; 4 Department of Economic Sociology, Faculty of Economics, İstanbul University, İstanbul, Turkiye

**Keywords:** Artificial intelligence, medicine, society-in-the-loop, bias, ChatGPT, accountability

## Abstract

The aim of this study is to examine the risks associated with the use of artificial intelligence (AI) in medicine and to offer policy suggestions to reduce these risks and optimize the benefits of AI technology. AI is a multifaceted technology. If harnessed effectively, it has the capacity to significantly impact the future of humanity in the field of health, as well as in several other areas. However, the rapid spread of this technology also raises significant ethical, legal, and social issues. This study examines the potential dangers of AI integration in medicine by reviewing current scientific work and exploring strategies to mitigate these risks.

Biases in data sets for AI systems can lead to inequities in health care. Educational data that is narrowly represented based on a demographic group can lead to biased results from AI systems for those who do not belong to that group. In addition, the concepts of explainability and accountability in AI systems could create challenges for healthcare professionals in understanding and evaluating AI-generated diagnoses or treatment recommendations. This could jeopardize patient safety and lead to the selection of inappropriate treatments. Ensuring the security of personal health information will be critical as AI systems become more widespread. Therefore, improving patient privacy and security protocols for AI systems is imperative.

The report offers suggestions for reducing the risks associated with the increasing use of AI systems in the medical sector. These include increasing AI literacy, implementing a participatory society-in-the-loop management strategy, and creating ongoing education and auditing systems. Integrating ethical principles and cultural values into the design of AI systems can help reduce healthcare disparities and improve patient care. Implementing these recommendations will ensure the efficient and equitable use of AI systems in medicine, improve the quality of healthcare services, and ensure patient safety.

## 1. Introduction

AI and automation are spreading to all areas of life. Especially with the continuously accumulating data, the scope of dissemination of these systems is expanding every day [1–4]. AI applications are becoming widespread from education to health, from defense industry to economy, from transportation to genetic research. Particularly, generative AI systems supported by large language models such as ChatGPT are now accessible, and they have the potential to fundamentally change daily life practices and thus culture [5].

The most profound impact of AI systems is directly felt in labor markets and indirectly in education systems [6,7]. The widespread adoption of AI systems and automation in all areas leads to dramatic transformations in the skill sets expected from conventional occupations. This transformation is predicted to render many occupations obsolete in the labor market while some occupations may survive with new skill sets, and it is also noted that new job positions previously not taught are emerging [8–10]. The direct impact of AI systems on transforming professions in labor markets naturally leads education systems to face the necessity of rapidly responding to this new and dynamic process [7]. While education systems strive to find solutions on how to benefit from AI systems in education, they are also confronted with the challenging task of making necessary changes in education curricula according to new skill sets and training teachers and academics accordingly [11–14].

The rapid development of AI systems supported by machine learning and deep learning is opening up new applications in the field of healthcare. Especially, the accumulation of big data in healthcare is accelerating the proliferation of AI systems in the healthcare domain [15]. The widespread use of image processing and classification, along with the significant advantages it provides, has accelerated the use of AI systems in fields such as radiology, pathology, gastroenterology, and ophthalmology, which rely heavily on image analyses [16]. Particularly, there is a widespread application area in the detection of cancer and monitoring of risky conditions related to cancer [17–20]. Similarly, AI systems can produce pathological interpretations faster, more accurately, and more conveniently compared to conventional approaches [16]. Additionally, they are widely used in improving risk assessment [21–23].

On the other hand, especially with the development and continuous updates of ChatGPT, AI systems now offer significant opportunities in preparing for medical education and competency exams [22,24]. ChatGPT is also used in preparing clinical notes by providing daily raw information about patients [25]. The features of ChatGPT such as text generation, summarization of articles and reports, rephrasing, and translation increase the likelihood of its use in essay, scientific report, and article preparation every day [26]. In fact, there is a debate about whether ChatGPT can be a coauthor in an article [26–30].

The rapid integration of AI systems into societies has shown that they carry risks as well as benefits. In particular, there is intense debate about how these systems can reproduce inequalities based on different characteristics such as socioeconomic status, race, and sex in society. They can even exacerbate inequalities and make them persistent, through the training dataset and modeling approaches used in algorithms [31–33]. On the other hand, ethical discussions, especially in the context of autonomous vehicles and defense industry applications, trigger debates on how AI systems can be developed by considering societal values and ethical principles not only in these areas but also in the design and implementation of all AI systems [34–39].

If used properly, AI has the potential to have a significant impact on the future of humanity in health and many other areas. However, the rapid proliferation of this technology raises critical ethical, legal, and social issues. This study addresses the potential dangers of integrating AI into medicine by reviewing the existing scientific literature and exploring strategies to mitigate these risks. AI is developing at an extraordinary rate. One of the main risks of AI in healthcare today is the quality of data sets. In any field, including health, the accuracy of AI’s decisions depends on the accuracy of its data. AI systems can also suffer from bias and exacerbate existing disparities in socioeconomic status, race, ethnicity, religion, sex, or disability [40,41]. While AI improves the accuracy of medical decision-making, it also introduces a high degree of uncertainty. For this reason, accountability is one of the most important risk areas in healthcare in the coming period. AI systems work with large amounts of data. With the widespread use of AI in healthcare, the privacy and security of personal data collected in healthcare will be another important area of concern. Therefore, it is essential to improve patient privacy and security protocols. The growing application of AI in healthcare has brought technological breakthroughs to traditional diagnosis and treatment. However, it also brings many risks and challenges. The quality of medical data will directly affect the quality of medical AI algorithm models. Algorithmic bias can affect the clinical predictions of AI. Lack of transparency in algorithms affects patient and physician trust in medical AI, and algorithmic errors or vulnerabilities can pose significant risks and harm to patients. The integration of medical AI into clinical practice can threaten the autonomy and dignity of physicians and patients. When accidents occur with medical AI, liability is unclear. All these factors affect people’s trust in medical AI [15]. There are many examples of applications of AI systems in healthcare as mentioned before such as image processing and classification, cancer detection and monitoring of high-risk conditions and health risk assessment and planning. For example, AI-powered risk assessment models are being used in processes to identify and manage high-risk patients [21,23]. As in many areas of education, AI offers important opportunities in medical education. For example, generative AI applications such as ChatGPT and Google Gemini provide personalized training and adaptive feedback to medical students [22,42]. The rapid integration of AI systems into health care comes with both benefits and risks. The increasing use of AI systems in education and medical applications requires necessary changes in educational curricula and teacher training programs [43].

Moreover, the preference for the automation path over the human-complementary path in the utilization of current AI systems in the labor market carries the risk of shifting the balance between human and machine in labor markets towards machines, thereby increasing unemployment, eroding societal harmony and resilience, and exacerbating inequalities to a greater extent [44]. Therefore, as expressed by Verhoeven et al. [26], ‘The problem of our time is not artificial intelligence, but what humans do with it’. In other words, the societal outcomes of AI systems will be determined more by our policies on how we deploy them rather than by the AI systems themselves [45]. The impact of AI is a constantly evolving and highly dynamic field. Every day we witness a new capability of AI. However, there are still uncertainties about whether these effects will be positive or negative. Therefore, this study aims to evaluate the impact of AI in the health sector based on the existing literature and provide solutions to the existing problems by contributing to the knowledge in this field. Then, in this study, the risks that the use of AI systems in the field of medicine may entail are generally assessed, and the steps needed to mitigate these risks are comprehensively addressed.

## 2. Challenges

Academic literature demonstrates the potential of AI technologies to revolutionize the diagnostic process for diseases such as retinal diseases [46], lung cancer [17], skin cancer [47], and breast cancer [19]. On the other hand, there are many barriers to ensuring the equitable use of AI at all levels of society. The use of AI systems in healthcare is directly associated with access to individuals’ health data, making data protection and privacy paramount among the risks in this context [34]. Considering that 15% of global data breaches occurred in the healthcare industry in 2017 [15], this situation is even more critical in the healthcare domain.

The most prominent flaw of AI systems is their tendency to exhibit discriminatory and biased behaviors towards specific groups in society based on the data they use for learning. A similar situation applies to AI systems used in the healthcare field as well [16,41]. The narrow representation of training data to a specific demographic can lead to biases when the AI system is used outside of this demographic [31]. Similarly, an AI system trained on data obtained from high-level medical facilities may exhibit biases when used in lower level medical facilities, and a system trained solely on data from Western populations may introduce biases when diagnosing Asian individuals [15,48].

It has been shown that when images of dermatological lesions from predominantly white patients are used in the training set, the accuracy of AI systems in identifying lesions in darker-skinned patients significantly decreases [40]. The accuracy of AI systems in diagnosing skin cancer based on patients’ skin images may decrease, especially in the summer months due to variations in skin tones caused by sun exposure [49]. Similarly, AI systems used in the diagnosis of melanoma may exhibit bias when applied to dark-skinned individuals due to the limited number of dark-skinned medical images in the learning skin lesion dataset [50]. Similar measurement bias is inevitable for AI systems trained on such data, as seen in pulse oximeter results systematically overestimating oxygen saturation in nonwhite patients, leading to racial bias [51].

If the training set in the healthcare domain is predominantly specific to one sex, significant performance drops are observed when the other sex is used during the testing phase [52]. On the other hand, there is a disparity described as the 10/90 gap in the support and funding of health research, where 90% of the funds are allocated to the health problems of 10% of the global population, and a significant portion of this 90% impoverished population is female [51,53,54]. Therefore, the data produced in research supported by these funds represents a very small fraction of the global population. Hence, the scope and context of the data sets used in learning are critically important in reducing biases, especially in the diagnosis phase. In other words, just like in other fields, AI systems in healthcare are only as good as the data provided to them [15].

Biases are not only embedded in the training data set but can also arise from assumptions made during algorithm creation, modeling, weighting attributes, and potentially biased variables [15,51,55]. For instance, despite the use of a general data set, disparities in access to healthcare can result in underrepresentation of socioeconomically disadvantaged groups in the learning data set, leading to biases against these groups [56]. Especially in identifying at-risk groups requiring advanced care with AI systems, considering healthcare expenditures, those with easy access to healthcare are more likely to be identified as at-risk groups and benefit from more advanced healthcare services, while disadvantaged groups facing access issues are less likely to benefit in this regard [23,57].

When access to services is not equalized, algorithms can prevent individuals from benefiting from services despite being classified as part of the at-risk group by assigning more false negatives to those who have less access to healthcare [41]. In this context, it has been shown that racial biases are particularly pronounced, with whites benefiting much more from high-risk care than other racial groups. In fact, when inequalities are corrected, it has been demonstrated that the additional assistance black patients would receive from these services increases from 17.7% to 46.5% [23].

When differences in hardware and software used in healthcare facilities, as well as different standards used in data generation and labeling, are not taken into account, AI systems trained on this data can lead to biases in other applications [15]. Therefore, data alone is not sufficient; knowing in which context and process it was produced enhances the usefulness of the data.

One challenging aspect related to the use of AI systems in the healthcare field is the increasing uncertainty or difficulty in explaining how the algorithm reaches a conclusion, especially with the use of machine learning and deep learning techniques and the utilization of large datasets [15,58]. In this case, doctors may feel uncertain about AI systems whose results they cannot explain [59], which can negatively impact the treatment process. Additionally, it creates a separate paradox regarding the solution, where algorithms with the best performance are the least transparent, while fully explanatory algorithms are less accurate [15,60].

On the other hand, as the opposite of uncertainty, an overreliance on the results generated by AI systems in healthcare can pose a risk of gradual decline in clinicians’ skills over time [16]. However, considering that AI systems need continuous monitoring to produce correct behaviors, this risk not only reduces the possibility of correcting wrong behaviors but also negatively affects the skills of clinicians.

Additionally, after AI systems are deployed, they continue to learn from new data and their behavior can vary according to the new data [61]. Similar risks apply in the healthcare domain [62]. In this new scenario, the performance of AI systems can worsen, leading to clinical risks [15,63]. Performance declines of AI systems in healthcare often stem from data drift or concept drift [51]. Therefore, in the healthcare domain, certification of AI systems at just the initial stage is insufficient, and a continuously monitored and updated certification process becomes critically important [16].

On the other hand, especially generative AI systems like ChatGPT offer opportunities in education and demonstrate successful performance in medical licensing exams, similar to other educational fields, they also have the potential to open up new horizons in medical education [42,64]. Particularly, the proliferation of generative AI systems such as ChatGPT enables personalized education and adaptive feedback, enriching educational environments to provide an interactive experience with new content [7,43,65]. Furthermore, the scope of the impact of these systems, especially in text generation contexts, continues to expand in scientific research fields [27,42].

These new approaches bring along not only their advantages but also new challenges. For instance, they increase the risk of cheating, especially in online exams [24]. Moreover, the potential use of these systems as writing assistants leads to an increase in plagiarism issues in scientific articles, undermining academic integrity [29,66]. The inadequacy of existing platforms for plagiarism and similarity detection exacerbates the problem [27]. If the problem of plagiarism arises from using ChatGPT for text generation, another issue is whether ChatGPT can be evaluated as an authorship [26]. Views and attitudes stating that ChatGPT cannot be evaluated as an author are increasing [26,30,67].

In this context, another risk is that the results and texts produced by these systems may not always be accurate, up-to-date, or may contain biases [42,64,68]. Especially, generative AI systems such as ChatGPT can produce responses in a reasonable manner even for nonexistent or inaccurate information, a phenomenon known as hallucination in AI systems [25,45,69,70]. Athaluri et al. [71] attempted to evaluate the frequency of AI hallucination in a scenario created using ChatGPT, and in the analysis of the 178 references in the generated result, it was shown that 69 did not have a DOI and 28 did not exist. Emsley [72], who had a similar experience, stated that labeling the current situation as hallucination is incorrect, and the correct labeling is fabrication and falsification. Therefore, whether labeled as hallucination or fabrication and falsification, this structural problem inherent in ChatGPT poses a significant risk, especially in medicine and healthcare. When approaching the generated results without caution, there is a risk of widespread dissemination of misinformation and perpetuation of inequalities associated with biases.

## 3. Participatory society-in-the-loop management

In this section, recommendations are provided regarding how to minimize the risks associated with the use of AI systems in the healthcare field, maximize their benefits, and create a responsible ecosystem in the context of responsible AI.

In this context, respect for patient privacy and the protection of personal data are among the foremost priorities [15]. Rajpurkar et al. [16] suggest the use of a federated model centered around decentralizing data storage to reduce risks related to data protection. In the federated model, private data is not shared; instead, AI models are sent to institutions that possess this data for training purposes. The same approach is employed for data updates in AI systems. This prevents data from being centralized in one location, reducing the risk or distributing it among different institutions. Utilizing data from various healthcare institutions across different geographical regions and hierarchical levels as much as possible will not only ensure data protection but also enhance the quality and representativeness of training data, thereby reducing biases [15,51].

The proper functioning of AI systems in the healthcare domain is directly influenced by numerous parameters, ranging from modeling features to the training data set. Increasing the representativeness of the training data set is crucial in reducing the impact of biases. Therefore, users of AI systems should be aware of the data set on which the system was trained and should be cautious of potential biases in the results. Similarly, labeling deficiencies in data sets also lead to biases. In cases where labeling is challenging, unsupervised setups requiring less labeling are recommended to mitigate the problems caused by labeling deficiencies [16].

Biases arise not only from the representativeness of the training data set but also from the approaches used in modeling [51]. For instance, when healthcare expenditures are considered in modeling to identify high-risk patients and those in need of advanced care, the model may overlook disparities in access to healthcare. As a result, disadvantaged social groups with limited access to healthcare services may disproportionately benefit less from these services [23]. Therefore, beyond the training data set, the assumptions and weighting factors used in modeling should be determined in a way that does not exacerbate inequalities [51,55]. Hence, the most effective approach to reducing biases is to ensure unbiased modeling and to use higher quality data sets in training [32].

Approaches focusing on continuously monitoring the behavior of AI systems to ensure they produce results consistent with the intended goals are gaining momentum. For example, Rahwan [38] emphasizes that AI systems’ codes say very little about the behaviors they produce, highlighting the need for continuous monitoring of these systems’ behaviors to detect and correct deviations before they exhibit incorrect behavior. Thus, discrepancies stemming from modeling or the training set can be identified and rectified [61]. A similar approach is particularly emphasized in the healthcare domain [36]. Indeed, in healthcare, especially, observations related to biases have been identified by continuously monitoring the behaviors generated by such systems [23,40,41,49]. Continuous monitoring of AI system behaviors can also prevent them from producing risky outcomes and deviations, especially when faced with new data [16].

In addition to continuous monitoring of AI system behaviors, a participatory management approach will have the most lasting impact in preventing biases and deviations from expected behavior in the healthcare domain. In the development and use of AI systems, researchers, technology users, and groups affected by this technology participating together in a participatory approach will not only prevent negative or unexpected outcomes but also share the power and control in modeling and design [4,73]. As a result, modeling is created in a participatory manner, preventing the use of biased key variables in modeling, and biases in the learning dataset are minimized, ensuring the generation of behaviors aligned with the intended goals [37,51].

The first phase of participatory management has been experienced through the human-in-the-loop approach. This approach has been used in various fields, from labeling data for training AI systems to interactive machine learning approaches, from human-machine collaborative systems to human-robot interactions, to ensure more effective functioning of AI systems [38,74–76]. Rahwan [38] suggests advancing participatory management in the development and use of AI systems to the next phase by including broader groups affected by these systems in the ‘human-in-the-loop’ approach, thus adding the society-in-the-loop approach, thereby incorporating the social contract. Consequently, not only will biases be prevented, but these systems will also be ensured to produce behaviors that are compatible with societal values. In fact, the society-in-the-loop approach also encompasses the crowdsourcing approach, which has been developed earlier and allows for the consideration of social values in algorithms [4,77,78].

In the healthcare domain, the society-in-the-loop approach requires the collaboration of multiple groups such as AI system experts, doctors, healthcare system administrators, insurance companies, representatives from social security institutions, sociologists, lawyers, patients, etc., in the design, development, and use of AI systems, and continuous improvement until the system produces the desired behaviors. It is crucial to have representatives from groups that are particularly affected by biases and are least represented in the model within these groups [51]. Since the development of AI systems encompasses various processes, collaboration throughout the entire process, from problem design and determining the most suitable variables to algorithm training, testing, deployment in real-world settings, and subsequent monitoring, can yield the expected benefits when defined comprehensively and extensively [51]. Moreover, such collaborations can also enhance AI literacy and competencies concerning all aspects of AI system development [42].

This approach will not only ensure that AI systems operate more healthily and in line with the intended goals but also enable the sharing of responsibilities regarding these systems in the healthcare domain. In other words, when AI systems are developed and implemented through such a network of stakeholders, risks and responsibilities will also be shared by the network [15]. Consequently, algorithms perceived as black boxes will be relatively better understood in terms of how they reach medical outcomes, leading to increased explainability and accountability of AI systems. Moreover, this enhanced explainability may contribute to researchers gaining a better understanding of the underlying biological mechanisms of diseases [16]. On the other hand, this culture will also prevent clinicians in the healthcare domain from developing overly reliant behavior towards AI systems, which could have long-term negative consequences. This approach will strengthen the human-complementary path, which has the potential to lead to greater prosperity in society in the long term, particularly by enhancing the skills of doctors to be more effective in the human-machine balance and to provide higher quality healthcare [44,45].

The most significant supporter of the society-in-the-loop approach will be the increase in AI literacy [14]. Enhancing the literacy of all relevant stakeholders, not just doctors, regarding the benefits of AI systems in healthcare, the opportunities they provide, the issues they may cause, and ethical considerations, will not only increase the safety of AI system usage in healthcare but also raise societal awareness about AI systems. In this context, it is crucial, particularly for healthcare institutions, to prioritize education on the use of AI systems for doctors, as this awareness is essential for understanding the dimensions of their responsibilities [15].

Increasing AI literacy can enhance the benefits of using these systems in education and academic research while also mitigating the risks associated with plagiarism and academic integrity [30]. All stakeholders in education and research should be able to clearly identify how AI systems can be used in education and research, the benefits they can provide, their limitations, and the risks they pose. They should also be able to define ethical principles and boundaries to protect academic integrity collectively. This will guide the education and scientific community in aligning their direction with these principles [26,30,42,67].

Recently, the use of professional algorithm auditors to determine whether algorithms deviate from their intended behaviors has become more widespread. Especially considering the proliferation of journalists who function as auditors, assessing the societal impact of AI systems in journalism [79], promoting this approach as a professional profession, not only in journalism but also in other fields, particularly in public health, will contribute to this process.

In sum, the future of the world, including health, will depend on the direction in which AI develops and how it is used. The ethical use of AI in a way that benefits broad segments of society will be one of the most important areas of discussion. This is because the widespread use of AI and the increasing capabilities of AI applications may lead to job losses in healthcare, as in many other fields. In particular, the combination of AI and robotics may lead to the substitution of not only white-collar workers but also blue-collar workers who require physical strength. Therefore, we propose that the participatory society-in-the-loop management approach favors the human-complementary path over the automation path in the utilization of current AI systems in the health sector in order to minimize the unemployment, the weakness in societal harmony, resilience and equalities to a greater extent.

## 4. Discussion and conclusion

AI systems now directly affect all areas of daily life. As the use of AI systems increases, an AI ecosystem consisting of technosocial systems is emerging. In this ecosystem, machine-human interaction is increasing day by day, and it is even directly transforming daily life practices, in other words, culture.

A similar transformation is deeply felt in the field of medicine as well. AI systems, machine learning, and deep learning are widely used in imaging systems, disease diagnosis and treatment processes, risk assessment and planning, as well as in education and research. Especially, the recent development and accessibility of generative LLM models like ChatGPT deepen this impact and transformation. Therefore, this study highlights the risks posed by the use of AI systems in the medical field and discusses the steps that need to be taken to increase the benefits of these systems and reduce risks.

The most important and urgent step to be taken in this regard is to increase AI literacy among doctors, students, specialists, healthcare managers, health insurance managers, social security system managers, and patients in the field of medicine. Considering the profound transformations caused by AI systems in all areas of societal life, AI literacy is now critical not only in the medical field but also in terms of daily life skills. Just as digital literacy has emerged as the most important literacy in all processes from education to work environments, especially after the COVID-19 pandemic [80–81], a similar situation now applies to AI literacy as well. Therefore, emphasis should be placed on training to increase the AI literacy of all stakeholders in the health and medical field. Especially considering how rapidly the use of AI systems is becoming widespread in medical education and scientific research, these training sessions should be continuously organized for medical students, doctors, and hospital managers who actively use these systems. Particularly, raising awareness of the ethical boundaries in the use of these systems in education and research will prevent plagiarism and preserve academic integrity.

Considering the risks that AI systems in the healthcare field have posed so far, it is seen that the most effective approach to mitigate and minimize these risks is the participatory society-in-the-loop management approach. Thus, with the active participation of stakeholders throughout the design, development, implementation, and subsequent monitoring stages of AI systems in medicine, processes will be managed more rationally, biases will be minimized, and the intended behaviors of these systems will be ensured. As the explainability and accountability of these systems increase, control and responsibilities can also be shared. Consequently, the context in which each AI system is produced and used in the medical field can be better understood, and the human-machine relationship can be established on a more rational basis by being aware of the risks it may pose as well as the benefits it may provide. In particular, equipping doctors with new AI skills in the field of medicine, rather than completely replacing humans with machines, will increase the value of labor. This will enable the AI ecosystem to be developed following the human-complementary path.

Past experience shows that when technological progress is not organized to serve all social groups equally, its benefits are often limited to privileged classes. In a liberal market economy, the upper classes have rapid access to new technologies and can benefit significantly from them, while the lower classes do not enjoy these benefits to the same extent. This can be seen as a reflection of technological injustice [1]. The development of AI technology is currently in the hands of a small number of large companies. Such a powerful technology, which can have a profound impact on the future of humanity, should not be left to the initiative of a few profit-seeking companies. The role of governments is crucial. Legislation must be enacted to ensure that AI is available for the benefit of large segments of society in a wide range of areas, from healthcare to education. These laws should promote and support the ethical and fair use of AI.

In addition, the importance of international cooperation and coordination cannot be underestimated. By collaborating and sharing information on AI policies, countries can support the development and use of the technology on a global scale in a fair and ethical manner. This cooperation can ensure that the potential of AI is fully realized for the benefit of all humanity. In this context, the equitable distribution and use of AI across social strata is not only a technological issue, but also an ethical and political imperative. Governments, businesses, and international organizations must take responsibility and work together. In this way, everyone can benefit fairly from the opportunities offered by AI technologies and ensure that technological progress benefits all segments of society. It is therefore critical that policymakers promote inclusiveness and accessibility in health services. Again, governments should monitor developments in AI; they should promote the protection of service users’ privacy, the security of their data, and policies of openness and transparency. Otherwise, as highlighted above, AI algorithms will increase misinformation and inequalities in society.

In this context, universities, public institutions, and professional organizations can organize inclusive AI training and certification programs to promote equal access to AI. The Ministry of Health, in cooperation with universities, can accredit and disseminate these programs. In addition, online versions of the training programs can be used to increase accessibility. Decision-makers need to establish regulations on privacy and security issues that will arise from the use of AI in the health sector. Otherwise, personal data commercialized by AI companies may lead to privacy violations. Under the leadership of the Ministry of Health, data sharing and processing processes can be developed among health institutions, hospitals, and universities using distributed learning models, an AI training method that allows data to be processed on local devices without being sent to a central server. These projects can be disseminated, especially among public hospitals and university hospitals, by ensuring data security.

In addition, the establishment of institutions for continuous monitoring and feedback and the transparency of their work will reduce risks. For this purpose, it would be right to establish independent review mechanisms or to benefit from future independent review mechanisms. To implement all this more effectively, the implementation of society-in-the-loop AI policies by involving different layers of society will increase inclusiveness while reducing potential risks. Although the participation of professional associations, NGOs, and universities in the decision-making process is not a common practice in many countries, the adoption of a participatory approach in the decision-making process, considering the magnitude of the possible risks of AI, will promote the proper use of AI.
